# Therapeutic Potential of Repeated Intravenous Transplantation of Human Adipose-Derived Stem Cells in Subchronic MPTP-Induced Parkinson’s Disease Mouse Model

**DOI:** 10.3390/ijms21218129

**Published:** 2020-10-30

**Authors:** Hyunjun Park, Keun-A Chang

**Affiliations:** 1 Department of Health Sciences and Technology, Gachon Advanced Institute for Health Sciences and Technology (GAIHST), Gachon University, Incheon 21936, Korea; hyunjun1991@hanmail.net; 2 Neuroscience Research Institute, Gachon University, Incheon 21565, Korea; 3 Department of Pharmacology, College of Medicine, Gachon University, Incheon 21936, Korea

**Keywords:** Parkinson’s disease, 1-methyl-4-phenyl-1,2,3,6-tetrahydropyridine-induced Parkinson’s disease mice, human adipose-derived stem cells, dopamine neurons, brain-derived neurotrophic factor, glial cell-derived neurotrophic factor

## Abstract

Parkinson’s disease (PD) is the second most common neurodegenerative disease, which is clinically and pathologically characterized by motor dysfunction and the loss of dopaminergic neurons in the substantia nigra, respectively. PD treatment with stem cells has long been studied by researchers; however, no adequate treatment strategy has been established. The results of studies so far have suggested that stem cell transplantation can be an effective treatment for PD. However, PD is a progressively deteriorating neurodegenerative disease that requires long-term treatment, and this has been insufficiently studied. Thus, we aimed to investigate the therapeutic potential of human adipose-derived stem cells (hASC) for repeated vein transplantation over long-term in an animal model of PD. In 1-methyl-4-phenyl-1,2,3,6-tetrahydropyridine (MPTP)-induced PD model mice, hASCs were administered on the tail vein six times at two-week intervals. After the last injection of hASCs, motor function significantly improved. The number of dopaminergic neurons present in the nigrostriatal pathway was recovered using hASC transplantation. Moreover, the administration of hASC restored altered dopamine transporter expression and increased neurotrophic factors, such as brain-derived neurotrophic factor (BDNF) and glial cell-derived neurotrophic factor (GDNF), in the striatum. Overall, this study suggests that repeated intravenous transplantation of hASC may exert therapeutic effects on PD by restoring BDNF and GDNF expressions, protecting dopaminergic neurons, and maintaining the nigrostriatal pathway.

## 1. Introduction

Parkinson’s disease (PD) is a gradually progressive neurodegenerative disease characterized by motor function decline. The primary causes of PD are age, genetic factors, and environmental factors; however, the exact mechanism underlying its onset is poorly understood. On the other hand, the risk factors for PD are age, heredity, sex, and exposure to neurotoxins. PD symptoms include slowed movement (bradykinesia), rigid muscles, impaired posture and balance, and shaking [1,2,3]. These motor disorders are influenced by the loss of dopaminergic neurons projected from the substantia nigra (SN) to the striatum (ST) [4]. PD treatments include levodopa (L-DOPA), a precursor of dopamine (DA); catechol-*O*-methyltransferase (COMT) and monoamine oxidase-B (MAO-B) inhibitors to prevent DA degradation; and dopamine D2 receptor agonists [5,6,7,8]. However, these treatments only alleviate PD. Currently, there is no clear treatment for PD, and this is because the mechanism remains to be unknown.

A lot of stem cell therapy studies have been conducted on neurodegenerative diseases, such as Alzheimer’s disease, amyotrophic lateral sclerosis, and PD [9,10,11]. Among them, treatment using mesenchymal stem cells (MSCs) has gained attention due to its potential to differentiate from the mesodermal lineage; the use of MSCs is also free from ethical problems compared to induced pluripotent stem cells (iPSCs) and embryonic stem cells (ESCs) [12,13,14]. Human adipose-derived stem cells (hASCs) are relatively easy to obtain among MSCs and have minimal adverse effects [15]. Therefore, hASC transplantation treatment in various diseases, including PD, has been continuously studied. A previous study has reported that dopaminergic neuron-differentiated ASC transplantation on a PD model may be an effective treatment [16]. Moreover, when autologous ASCs were transplanted in a 6-hydroxydopamine (6-OHDA)-induced PD rat model, the dopaminergic neuron was restored, microglial activation was decreased, and motor function was improved [16]. However, another study demonstrated that hASC transplantation in a 6-OHDA-induced PD rat model reduced the degeneration of dopamine neurons in SN but did not improve motor function [17]. When hASCs were transplanted in the vein of a 6-OHDA-induced PD animal model, mitochondrial function was restored [18]. These results suggest that ASCs may be potentially effective in treating PD. However, no observational study has been performed on the efficacy of repeated transplantation of hASCs in a PD mouse model.

Among various toxic PD models, the 1-methyl-4-phenyl-1,2,3,6-tetrahydropyridine (MPTP) model is the most commonly used [19]. MPTP, a precursor of MPP+, is a neurotoxin that produces a reliable and reproducible lesion of the nigrostriatal dopaminergic pathway after its systemic administration [20,21]. The MPTP mouse model faithfully reproduces neurodegeneration naturally occurring in PD. The most common, reliable, and reproducible lesion is caused by the systemic subcutaneous or intraperitoneal cavity administration. In the MPTP model, the magnitude of nigrostriatal damage depends on the dose and dosing schedule [20].

On the one hand, acute models are unsuitable for distinguishing the long-term effects of hASCs, because DA neurons recover over time and many dopamine neurons die within 24 h, which are both known to exhibit high mortality [22]. On the other hand, in chronic models, dopamine neuronal loss is maintained for a long time, but MPTP must be continuously injected at four- to five-day intervals or administered using devices, such as osmotic pressure [23], which can directly affect the hASCs administered multiple times into the tail vein. In the subchronic PD model, apoptosis was observed in the SN but did not exhibit mortality nor lose dopaminergic neurons in the SN. Therefore, we investigated the efficacy of PD treatment with repeated transplantations of hASC over a long-term in a subchronic MPTP-induced PD model.

## 2. Result

### 2.1. hASCs Alleviate Motor Deficits in MPTP-Induced PD Mice

**Figure 1:**
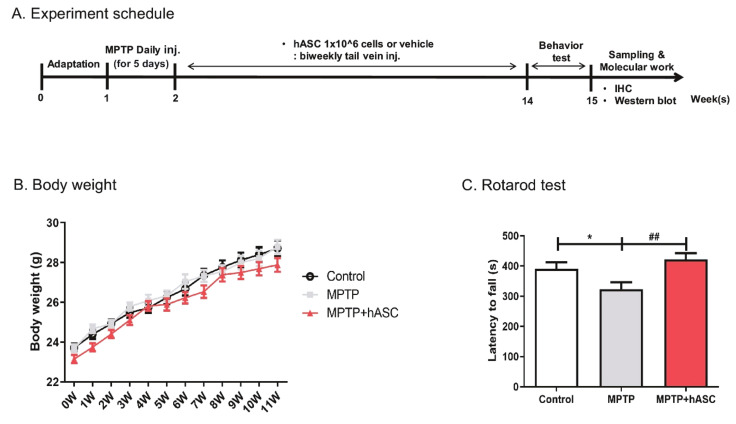
Effects of human adipose-derived stem cells (hASC) transplantation on motor function in 1-methyl-4-phenyl-1,2,3,6-tetrahydropyridine (MPTP)-induced Parkinson’s disease mice. (**A**) Experimental schemes: MPTP was injected into the abdominal cavity of mice at a concentration of 30 mg/kg for 5 consecutive days. One week after MPTP injection, hASCs (1 × 10^6^ cells/100 μL) were administered into the tail vein 6 times at 2-week intervals. Behavioral tests were performed 2 weeks after the last hASC transplant, and the mice were sacrificed for brain tissue sampling. (**B**) The weight of the mice was measured weekly during hASC transplantation, which did not differ between groups. (**C**) The latency time in the rotarod performance test was decreased in MPTP mice compared to that in control mice and significantly increased in the MPTP + hASC mice. All data are indicated as mean ± standard error (*n* = 9 mice per group). * *p* < 0.05 vs. Control and ## *p* < 0.01 vs. MPTP using one-way analysis of variance (ANOVA) and Bonferroni’s post hoc multiple comparisons test.

To induce the PD animal model, MPTP was consecutively injected into the abdominal cavity for five days (Figure 1A). The rotarod test was performed before and after MPTP administration to check whether a PD animal model had developed. There was no difference in waiting time before MPTP administration, but the waiting time after MPTP administration was decreased compared to the control (Figure S1A,B). After MPTP injection, vehicle or hASCs were administered into the tail vein six times at two-week intervals. This study involved three groups of mice: vehicle-treated control (control) group, vehicle-treated MPTP (MPTP) group, and hASC-treated MPTP (MPTP + hASC) group. The groups did not differ in terms of body weight during the 12 weeks of hASC transplantation (Figure 1B). Following the last hASC transplantation, a rotarod test was performed to investigate whether the motor function was alleviated. Consequently, MPTP mice demonstrated a significant decrease in latency time compared to the control mice, which was significantly recovered by hASC transplantation (control, 398.1 ± 20.99; MPTP, 310.6 ± 16.53; *p* < 0.05 vs. control; MPTP + hASC, 422.4 ± 19.79; *p* < 0.001 vs. MPTP; F (2, 24) = 9.378, *p* = 0.0010) (Figure 1C). In the pole test, the latency time in MPTP mice increased compared to the control mice and significantly decreased in the MPTP + hASC mice (Figure S1C).

### 2.2. hASCs Alleviate TH-Positive Cells in MPTP-Induced PD Mice

**Figure 2:**
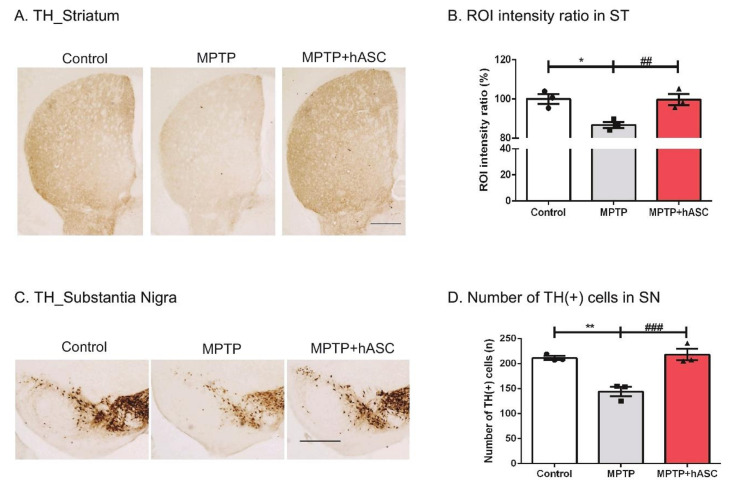
Effects of hASC transplantation on the loss of tyrosine hydroxylase (TH)-positive dopaminergic neurons in the striatum and substantia nigra of MPTP-induced Parkinson’s disease mice. The brain slices were immunostained using an anti-TH antibody in the striatum and substantia nigra regions. TH-positive dopaminergic neurons in the striatum and substantia nigra were microscopically visualized using diaminobenzidine (DAB) stain. (**A**) Representative images of TH immunoreactivity in the striatum from mice. Scale bars, 200 μm. (**B**) The average intensity of TH-positive cells in the striatum was illustrated using a graph. (**C**) Representative images of TH immunoreactivity in the substantia nigra from mice. Scale bars, 200 μm. (**D**) The average number of TH-positive cells in the substantia nigra were counted and illustrated using a graph. Dopaminergic neuronal cell death in the striatum and substantia nigra regions sharply increased following the injection of MPTP, and dopaminergic neurons recovered with hASC transplantation. All data are indicated as mean ± standard error (*n* = 3 per group). * *p* < 0.05 and ** *p* < 0.01 vs. Control, and ## *p* < 0.01, ### *p* < 0.001 vs. MPTP using one-way ANOVA and Kruskal–Wallis multiple comparisons test.

After the motor function test, we performed immunohistochemical analysis using the tyrosine hydroxylase (TH) antibody to examine the recovery of dopaminergic neurons in the ST (Figure 2A) and SN (Figure 2C). In the ST, TH intensity significantly reduced in MPTP-treated mice compared to that in control mice but recovered in MPTP + hASC mice (control, 100.00 ± 2.924; MPTP, 86.78 ± 1.364; *p* < 0.05 vs. control; MPTP + hASC, 99.75 ± 2.213, *p* < 0.01 vs. MPTP; *p* = 0.0006) (Figure 2B). In the SN, the number of TH-positive cells was determined. There were significantly fewer TH-positive cells observed in the MPTP group than in the control group, but they were highly recovered in the MPTP + hASC group (control, 211.3 ± 12.54; MPTP, 144.3 ± 10.13; *p* < 0.01 vs. control; MPTP + hASC, 217.9 ± 13.64; *p* < 0.001 vs. MPTP; *p* = 0.0006) (Figure 2D). Furthermore, the number of TH-positive cells was measured in the ventral tegmental area (VTA) region. Consequently, there was no difference between groups (Figure S2C).

**Figure 3:**
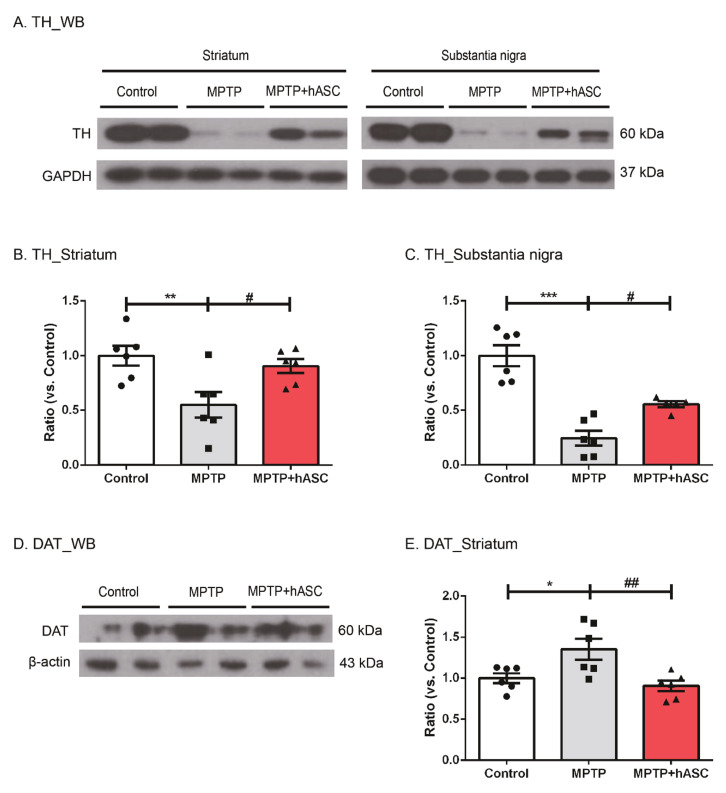
Effects of hASC transplantation on dopaminergic neurons and dopamine transporter in MPTP-induced Parkinson’s disease mice. Western blotting was performed on the brain tissues of striatum and substantia nigra regions in each group using anti-TH or DAT antibodies. (**A**) Representative Western blots of TH in the striatum and substantia nigra tissue lysates in each group using TH and GAPDH antibodies. (**B**,**C**) The graph was expressed as the ratio of TH in the striatum (**B**) and substantia nigra (**C**) normalized with GAPDH (*n* = 6 per group). (**D**) Representative Western blots of dopamine transporter (DAT) in the striatum tissue lysates of each group using DAT and β-actin antibodies. (**E**) The graph was expressed as the ratio of DAT in the striatum normalized using β-actin. All data are indicated as mean ± standard error (*n* = 6 per group). * *p* < 0.05, ** *p* < 0.01, *** *p* < 0.001 vs. Control, and # *p* < 0.05, ## *p* < 0.01 vs MPTP using one-way ANOVA and Bonferroni’s post hoc multiple comparisons test.

Furthermore, we performed Western blotting using TH antibody to confirm the recovery of dopaminergic neurons in the ST and SN (Figure 3A). Regarding the ST, the level of TH expression markedly decreased in MPTP mice compared to that of control mice; however, hASC transplantation significantly restored the level of TH (control, 1.000 ± 0.089; MPTP, 0.550 ± 0.119; *p* < 0.05 vs. control; MPTP + hASC, 0.906 ± 0.063; *p* < 0.05 vs. MPTP; F (2, 15) = 6.489, *p* = 0.0093) (Figure 3B). Moreover, in the SN, the level of TH expression decreased in MPTP mice compared to that of control mice but recovered in MPTP + hASC mice (control, 1.000 ± 0.095; MPTP, 0.245 ± 0.067; *p* < 0.0001 vs. control; MPTP + hASC, 0.554 ± 0.028; *p* < 0.05 vs. MPTP; F (2, 14) = 28.54, *p* < 0.0001) (Figure 3C). To elucidate the basic mechanism underlying motor function recovery by hASC transplantation, we evaluated the expression of dopamine transporter (DAT) in the ST (Figure 3D). In MPTP mice, DAT expression increased compared to that of control mice but decreased in MPTP + hASC mice (control, 1.000 ± 0.060; MPTP, 1.353 ± 0.127; *p* < 0.05 vs. control; MPTP + hASC, 0.907 ± 0.062; *p* < 0.01 vs. MPTP; F (2, 15) = 7.037, *p* = 0.0070) (Figure 3E).

### 2.3. hASCs Increase the Levels of BDNF and GDNF Expression in MPTP-Induced PD Mice

**Figure 4:**
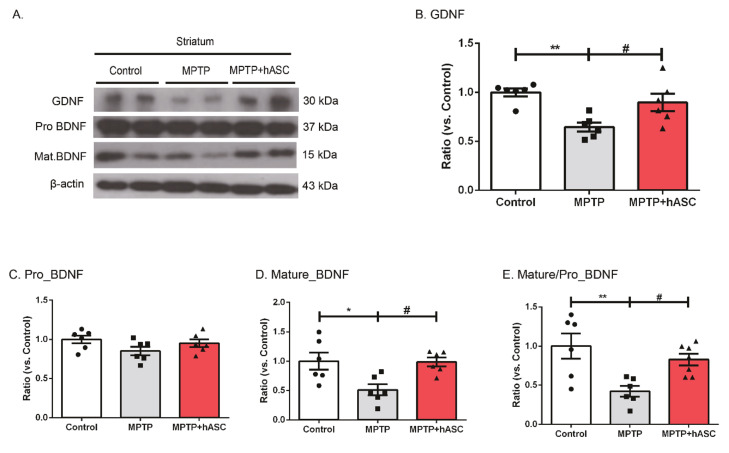
Effects of hASC transplantation on brain-derived neurotrophic factor (BDNF) and glial cell-derived neurotrophic (GDNF) expression in the striatum of MPTP-induced Parkinson’s disease mice. Western blotting analysis was performed using brain tissues of the striatum and substantia nigra regions in each group using anti-BDNF or GDNF antibodies. (**A**) Representative Western blots of TH in the striatum tissue lysates of each group using BDNF, GDNF, and GAPDH antibodies (*n* = 6 per group). (B–E) The graph was expressed as the ratio of GDNF (**B**), pro-BNDF (**C**), mature BDNF (**D**), and mature/pro-BDNF, and (**E**) normalized using β-actin internal control. All data are indicated as mean ± standard error (*n* = 6 per group). * *p* < 0.05 and ** *p* < 0.01 vs. Control, and # *p* < 0.05 vs. MPTP using one-way ANOVA test and Bonferroni’s multiple comparisons test.

To grasp the mechanism underlying dopaminergic neuronal recovery by hASC transplantation, protein levels of neurotrophic factors, such as the brain-derived neurotrophic factor (BDNF) and glial cell-derived neurotrophic factor (GDNF), were evaluated using Western blotting (Figure 4A). The level of GDNF significantly decreased in the ST of MPTP mice compared to that in control mice but recovered in MPTP + hASC-treated mice (control, 1.000 ± 0.039; MPTP, 0.646 ± 0.045; *p* < 0.01 vs. control; MPTP + hASC, 0.897 ± 0.088; *p* < 0.05 vs. MPTP; F (2, 15) = 8.799, *p* = 0.0030) (Figure 4B). Pro-BDNF showed no statistical differences in all groups (control, 1.000 ± 0.050; MPTP, 0.852 ± 0.055; MPTP + hASC, 0.9525 ± 0.050; F (2, 15) = 2.106, *p* = 0.1563) (Figure 4C). However, the level of mature BDNF significantly decreased in MPTP mice compared to that in control mice but significantly recovered in MPTP + hASC mice (control, 1.000 ± 0.147; MPTP, 0.512 ± 0.094; *p* < 0.05 vs. control; MPTP + hASC, 0.988 ± 0.076; *p* < 0.05 vs. MPTP; F (2, 15) = 6.450, *p* = 0.0095) (Figure 4D). Furthermore, the ratio of mature to pro-BDNF in MPTP mice was lower than that in control mice but higher in MPTP + hASC mice than in control mice (control, 1.000 ± 0.161; MPTP, 0.422 ± 0.068, *p* < 0.01 vs. control; MPTP + hASC, 0.829 ± 0.073; *p* < 0.05 vs. MPTP; F(2, 16) = 7.436, *p* = 0.0052) (Figure 4E).

## 3. Discussion

In this study, we discovered that intravenous injections of hASCs in MPTP-induced PD model mice alleviated motor function by restoring the levels of BDNF and GDNF expression in dopaminergic terminals.

Animal models primarily used in PD studies include genetically modified (transgenic) models and toxin (e.g., 6-OHDA and MPTP)-induced models. The transgenic models accumulate alpha-synuclein, a hallmark of PD; however, it is challenging to identify motor dysfunction. The 6-OHDA-induced PD model is developed by injecting 6-OHDA directly into the SN to destroy dopaminergic neurons, which primarily exhibit the characteristics of end-stage PD. MPTP-induced models are relatively easy to develop, and they exhibit motor dysfunction and dopamine neuronal cell death of animal models that administer MPTP into the subcutaneous or intraperitoneal cavity. Besides, the loss of ancestral dopamine persists for one month after the last administration of neurotoxins [24]. In this study, the MPTP-induced model was selected to confirm motor function and dopaminergic neuronal cell recoveries by hASC transplantation.

Currently, studies on the treatment of various diseases using MSCs are actively being conducted. MSCs can be relatively easy to isolate and are free from ethical problems; they can also be developed into multipotent and differentiated cell types. Among MSCs, hASCs can be classified into various types, such as muscle, liver, cardiac, and nerve. Moreover, ASCs have no adverse effects, such as tumor formation, chromosome abnormalities, and immune rejection, and are safe for hASC transplantation in animals and humans [15]. Therefore, research on PD treatment using hASCs is ongoing. Preclinical studies have demonstrated that dopamine neurons recovered and motor function improved when hASCs were transplanted into neurotoxin-induced PD models [17,18,25]. Based on these studies, considerations for cell-based clinical trials for PD are currently being discussed [26,27,28,29]. However, most studies only reported a short-term effect following ASC administration, once or twice and intravenously or directly in the SN.

The most convenient method of MSC administration is intravenous injection. However, only ≤1% of transplanted cells move to the target site [30]. MSC green fluorescent protein (GFP)-luciferase was injected into the SN of mice. After 5 days, the GFP expression was reduced by almost 50% [31]. Furthermore, fluorescence microscopy revealed no detectable GFP-positive cells in the brain at 4 weeks after transplantation [32]. This proves that transplanted MSCs are delivered to the brain in small amounts and remain there only for a short time. Thus, we transplanted hASCs once every 2 weeks for 12 weeks, so that the efficacy of the transplanted hASCs can be maintained. We performed immunohistochemical analyses using human neutrophil antigen antibodies to confirm that the transplanted hASCs migrated to the brain, and we found that a very small number of human-specific nuclei in the hASC-transplanted brains (Figure S5). Our results suggest that repeated intravenous administration of hASCs can restore impaired motor function and recover dopaminergic neurons and dopamine transporters.

However, the therapeutic mechanism of hASC transplantation remains unclear, but it is believed that the secretome secreted by hASC significantly contributes to the therapeutic effect in disease models. Various growth factors and cytokines have been identified in the secretome secreted by hASCs. Several neurotrophic factors (NTF) secreted from MSCs, including BDNF and GDNF, are known to affect the neuroprotective and neurorestorative effects of dopaminergic neurons [33]. Increased NTF expression serves as neuroprotection against brain damage, protecting neurons against oxidative stress, excitotoxicity, and apoptosis [34]. In previous studies, BDNF administration in PD animal models inhibited the loss of dopaminergic neurons in the ST and SN [35,36,37,38]. Recent studies have also reported that GDNF exerts positive effects on motility and promotes the survival of intracranial neural stem cells in PD [39,40]. Here, we demonstrated that BDNF and GDNF expressions increased in MPTP-induced PD mice that were intravenously injected with hASCs. Although it was not confirmed whether the administered hASCs were differentiated into dopamine neurons after migrating to the SN or ST, secretome secreted from the administered hASCs may have activated the expression of BDNF and GDNF.

Ultimately, we revealed that repeated intravenous transplantation of hASCs improved motor deficits in MPTP-induced PD mice. Although our results indicated that it was mediated by reduced dopamine neuronal cell death via increased NTF, such as BDNF and GDNF, further studies are warranted to fully elucidate the mechanisms involved. We believe that hASCs may be a useful therapeutic agent for the treatment of neurodegenerative diseases, including PD.

## 4. Materials and Methods

### 4.1. Production of MPTP-Induced PD Mouse Model and hASC Administration

Seven-week-old C57BL/6J male mice were purchased from DBL (Eumseong, Korea). The animals were maintained under standard conditions (12⁄12-h light/dark cycle, 21 ± 2 °C) and relative humidity of 40% with ad libitum access to food and water. For the PD model, MPTP was injected into the abdominal cavity at a concentration of 30 mg/kg for 5 consecutive days. hASCs were provided by the Biostar Stem Cell Research Institute on the day of injection and were immediately administered upon receipt. One week after MPTP injection, hASCs (1 × 10^6^ cells/100 μL) were administered into the tail vein 6 times at 2-week intervals. The hASC study was approved by the Committee of Biostar Stem Cell Research Institute (RBIO 2017_02_001). Experimental groups were divided into control mice and MPTP-induced PD model mice treated with either vehicle (MPTP) or hASCs (MPTP + hASC), and each group consisted of 9 mice (*n* = 9 per group). All animal experiments were approved by the Institutional Animal Care and Use Committee of the Lee Gil Ya Cancer and Diabetes Institute, Gachon University (ICACU No. LCDI-2017-0119, 20 July 2018).

### 4.2. Behavior Test

After 6 hASCs injections, motor function was tested using the accelerating rotarod test. Before the test, all mice were conditioned to attain a speed of 5 rpm for 5 min to get used to the rotarod apparatus (B.S. Technolalb INC., Seoul, Korea). The day after the training, motor performance was evaluated at an accelerated speed from 5 rpm to 40 rpm for 10 min. The experiment was performed in each mouse for 3 trials, and each trial was rested for at least 10 min to relieve stress and fatigue. The rotarod test of each mouse was calculated by averaging the latency times measured in 3 trials.

The time schedule of the experimental procedure is illustrated in Figure 1.

### 4.3. Tissue Sampling

The mice were anesthetized using a zoletil (12.5 mg/kg) and rompun (17.5 mg/kg) mixture and transcardially perfused with saline-containing heparin. Furthermore, their brains were isolated for immunohistochemical and Western blot analyses. For immunohistochemistry, the brains were fixed using 4% paraformaldehyde at 4 °C for 24 h. Thereafter, they were dehydrated in 30% sucrose solution at 4 °C for 3 days. The brain tissues were frozen into optimal cutting temperature compound (Sakura Finetek, Tokyo, Japan), coronally sectioned into 20-μm-thick slices using cryotome (Thermo Fisher, Waltham, MA, USA), and stored in a cryoprotectant solution at 4 °C. For Western blotting, the ST and SN were dissected from non-perfused brains, snap-frozen in liquid nitrogen, and stored at −80 °C for later use.

### 4.4. Immunohistochemistry

The brain slides were recovered for 10 min at 60 °C with 3% hydrogen peroxide, washed in phosphate-buffered saline (PBS) containing 0.2% Triton X-100, and were then incubated in a blocking solution (0.5% bovine serum albumin and 3% normal horse serum in PBS with 0.4% Triton X-100) at room temperature (RT) for 1 h. The brain slices were incubated with the primary antibody in PBS containing 0.2% Tween 20 (PBS-T) at 4 °C overnight. After washing in PBS-T buffer, the brain tissues were incubated with a VECTASTAIN^®^ Elite ABC-HRP Kit Peroxidase (Rabbit IgG) (PK-6101; Vector Laboratories Inc., Burlingame, CA, USA) at RT for 1 h. For diaminobenzidine (DAB) staining, the brain slides were incubated with liquid DAB Substrate Kit chromogen (ab64238; Abcam, Cambridge, UK) at RT for 1–2 min. The slides were washed using PBS and coverslipped with the mounting solution (DAKO, Santa Clara, CA, USA). The ST and SN were imaged using an EVOS XL Core Imaging System (Thermo Fisher, Waltham, MA, USA). Forty times magnified images were captured on an average of 2–3 sections per animal that were 20 μm apart from each other. For measuring the TH-stained striata, each slide was compared and analyzed by region of interest intensity ratio (%) using the NIS-Elements 4.30.00 program (Nikon Corporation, Tokyo, Japan) (*n* = 3 per group). With regard to the number of TH-positive cells in the SN, similar SN regions per animal were selected, and 3 researchers blindly counted the number of TH-positive cells (*n* = 3 per group). Three researchers blindly quantified the values from captured images as the number of cells per area in the SN using Image J software v. 1.4.3.67 (NIH, Bethesda, MD, USA).

### 4.5. Western Blotting

Proteins were extracted from brain tissues using the radioimmunoprecipitation assay (RIPA) buffer (50 mM Tris pH 8.0, 150 mM NaCl, 1% NP-40, 0.1% SDS), and protein concentrations were measured using Bradford protein assays (Bio-rad, Hercules, CA, USA). Protein extracts (5–10 µg) were denatured at 95 °C for 10 min and separated using sodium dodecyl sulfate-polyacrylamide gel electrophoresis (SDS-PAGE). Proteins were transferred to a polyvinylidene difluoride (PVDF) membrane (Millipore, Burlington, MA, USA), and the membrane was blocked with 3% bovine serum albumin (BSA) in tris-buffered saline (TBS) containing 0.2% Tween 20 (TBS-T) at RT for 1 h. The membrane was incubated with a proper primary antibody (Table 1) in 3% BSA in TBS buffer at 4 °C overnight. After washing, the membrane was incubated with a proper secondary antibody (Table 1) at RT for 1 h. Signals from immunoblots were developed using the immobilon Western blotting horseradish peroxidase (HRP) conjugate (WBKLS0500; Millipore, Burlington, Massachusetts, USA) to visualize protein bands. Signals were detected using X-ray films. The density of the bands was analyzed using Image J software v. 1.4.3.67 (NIH, USA).

### 4.6. Statistics

All statistical analyses were performed using GraphPad Prism v. 8.4.2 (679) software (GraphPad Software Inc., San Diego, CA, USA), and outliers were removed using the Outlier calculator (significance level: Alpha = 0.05) in GraphPad Prism v. 8.4.2 (679) software (GraphPad Software Inc., San Diego, CA, USA). All data were indicated as the mean ± standard error. Differences of all collected data between groups were analyzed using one-way analysis of variance followed by the Bonferroni post hoc test, and Kruskal–Wallis was conducted by non-parametric groups. *p*-values of <0.05 were considered statistically significant (* *p* < 0.05; ** *p* < 0.01; *** *p* < 0.001; **** *p* < 0.0001).

## Figures and Tables

**Table 1:** Antibodies used in this study

Antibody	Company	Cat No.	Titer	Molecular Weight (kDa)	Source
TH (Immunohistochemical analysis)	Santa Cruz	sc-14007	1:200	-	Rabbit
TH (Western blotting)	Santa Cruz	sc-14007	1:2000	60	Rabbit
Recombinant Anti-BDNF	Abcam	ab108319	1:2000	15/37	Rabbit
Anti-GDNF antibody	Abcam	ab18956	1:2000	25	Rabbit
β-actin antibody	Santa Cruz	sc-47778	1:2000	43	Mouse
GAPDH (A531)	Bioworld	AP0066	1:3000	36	Rabbit
Goat anti-mouse IgG (H + L)-HRP conjugate	Bio-rad	#170-6516	1:10,000	-	Goat
Goat anti-rabbit IgG (H + L)-HRP conjugate	Bio-rad	#170-6515	1:2000~1:10,000	-	Goat

## Supplementary Materials

Supplementary materials can be found at https://www.mdpi.com/1422-0067/21/21/8129/s1.

## Abbreviations

BDNF	brain-derived neurotrophic factor
DAB	diaminobenzidine
DAT	dopamine Transporter
GAPDH	glyceraldehyde-3-phosphate dehydrogenase
GDNF	glial cell-derived neurotrophic factor
hASCs	human adipose-derived stem cells
MPTP	1-methyl-4-phenyl-1,2,3,6-tetrahydropyridine
MSC	mesenchymal stem cells
NTF	neurotrophic factor
PD	Parkinson’s disease
SN	substantia nigra
ST	striatum
TH	tyrosine hydroxylase
