# Supplementary material for: Therapeutic Potential of Repeated Intravenous Transplantation of Human Adipose-Derived Stem Cells in Subchronic MPTP-Induced Parkinson’s Disease Mouse Model
Source: Int J Mol Sci. 2020 Oct 30;21(21):8129. doi: 10.3390/ijms21218129 (PMC7663651; doi:10.3390/ijms21218129)
Supplement: Supplementary file 1 [file ijms-21-08129-s001.pdf]

Supplementary Materials and Methods

Pole test

The motor coordination was tested using a pole test. A vertical wood pole which was 50 cm in height and 1cm in diameter was placed in the home cage. Mice placed head-up on top of the pole, orient themselves downward and descend the pole back into the home cage. The head of the mice was directed toward the top of the pole, and then measured the total time to climb down to the home cage. The experiment was measured for 1 minute, and it was performed five times in total, and at least 5 minutes of rest was given between each experiment. All experiments were recorded using Ethovision XT 9 software (Noldus, Netherlands). The results of this study were used as the averages of the three fastest descending times. The pole test was performed by five per each group.

Immunohistochemistry

Brain slides were washed in phosphate-buffered saline (PBS) containing 0.2% Triton X-100, and were then incubated in a blocking solution (0.5% bovine serum albumin and 3% normal horse serum in PBS with 0.4% Triton X-100) at room temperature (RT) for 1 h. The brain slices were incubated with HNA antibody (1:200; MAB1281; Millipore, Burlington, MA, USA) in PBS containing 0.2% Tween 20 (PBS-T) at 4℃ overnight. After washing in PBS-T buffer, the brain tissues were then incubated with Alexa Fluor™ 555 goat anti-mouse IgG (H+L) antibody (A21422; Invitrogen, Carlsbad, CA, USA) at RT 1h. The slides were washed with PBS and coverslipped with the mounting solution (Millipore, Burlington, MA, USA). The brain imaged were captured by a Zeiss LSM 700 confocal microscope (Oberkochen, Germany) with a 10x objective and optical zoom 4x.

Supplementary Figures

A. Rotarod test

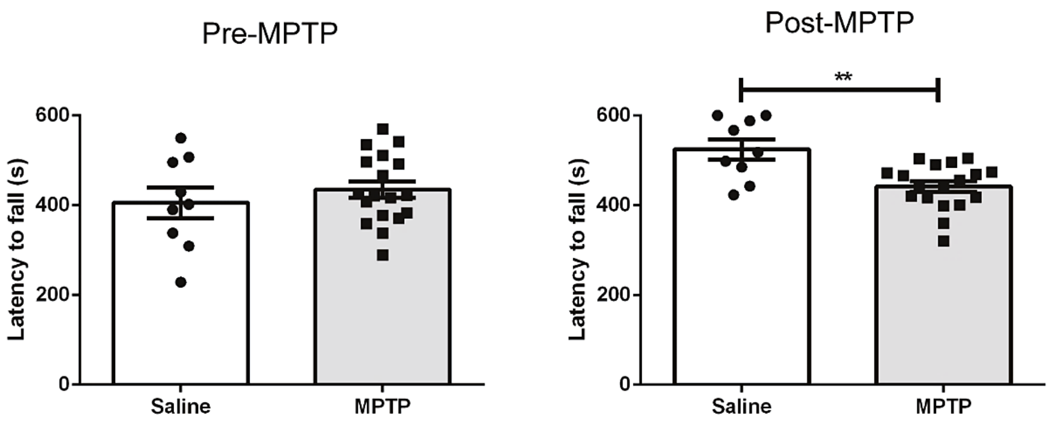

B. Pole test

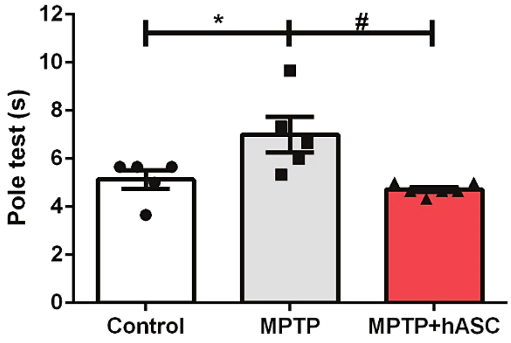

Supplementary Figure 1.

(A) The latency time in the Rotarod performance test was not different between control mice (n = 9) and MPTP mice (n = 18) before MPTP injection. The latency time in the Rotarod performance test was decreased in MPTP mice (n = 18) compared to control mice (n = 9) after MPTP injection. (B) The latency time in the pole test was increased in MPTP mice compared to control mice and significantly decreased in the MPTP + hASC mice (n = 5 per group). All data are indicated as the mean ± standard error. *$p < 0.05$ and **$p < 0.01$ by one-way analysis of variance (ANOVA) and Bonferroni posthoc multiple comparisons test.

A. TH_Striatum

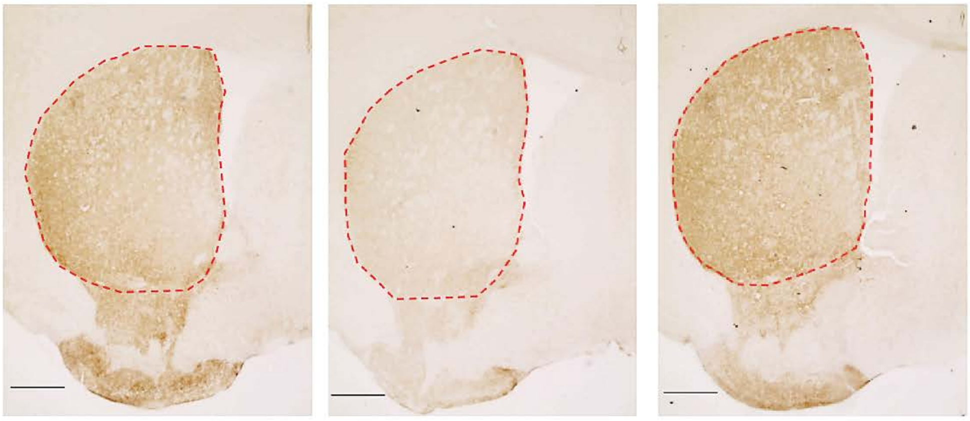

B. TH_Substantia nigra

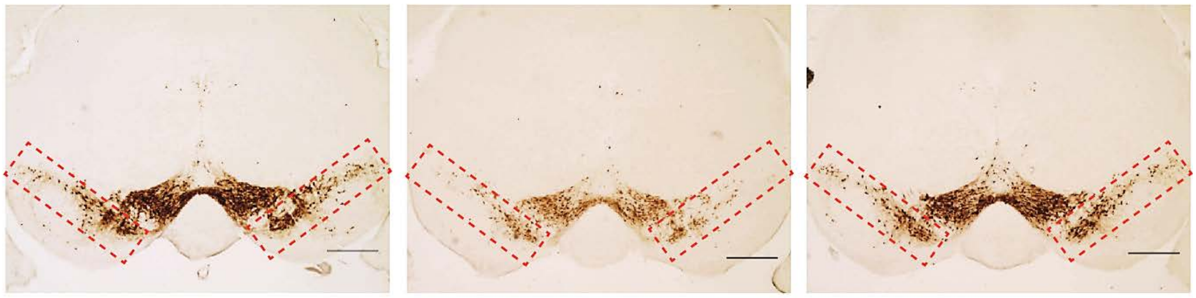

C. Number of TH(+) cells in VTA

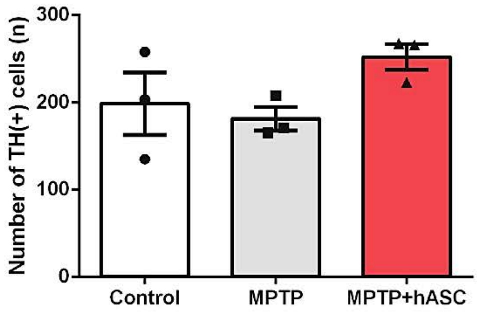

Supplementary Figure 2. IHC images of the striatum (A) and substantia nigra (B) used for quantification of Figure 2. TH-positive cells in the area indicated by the red dotted line were analyzed. (C) The average intensity of TH-positive cells in the VTA was illustrated by a graph. All data are indicated as mean ± standard error (n = 3 per group). One-way analysis of variance (ANOVA) and Kruskal-Wallis multiple comparisons test.

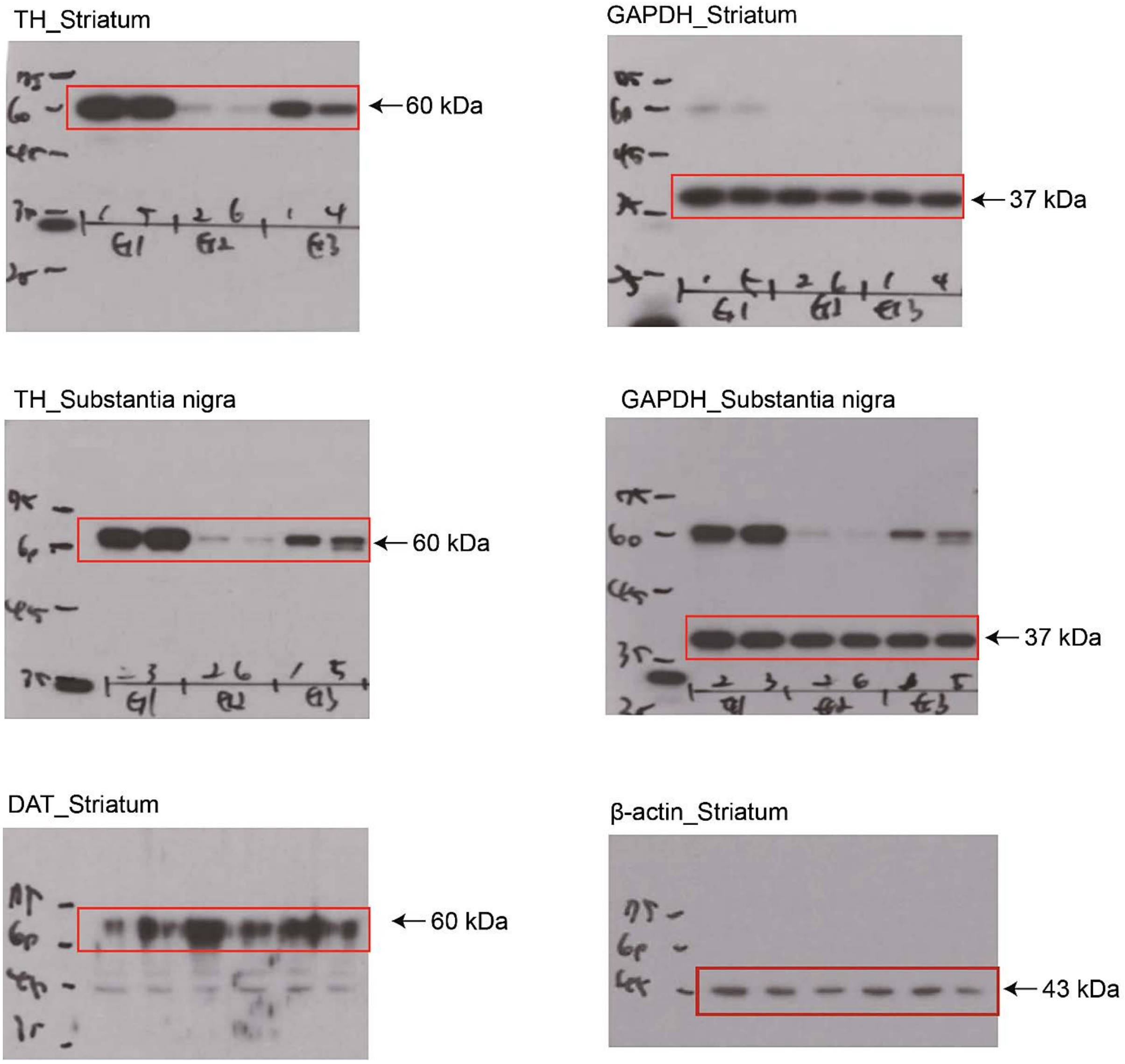

Supplementary Figure 3. Full blots of Figure 3. We read the bands in the red square as TH normalized by GAPDH and DAT normalized by β-actin.

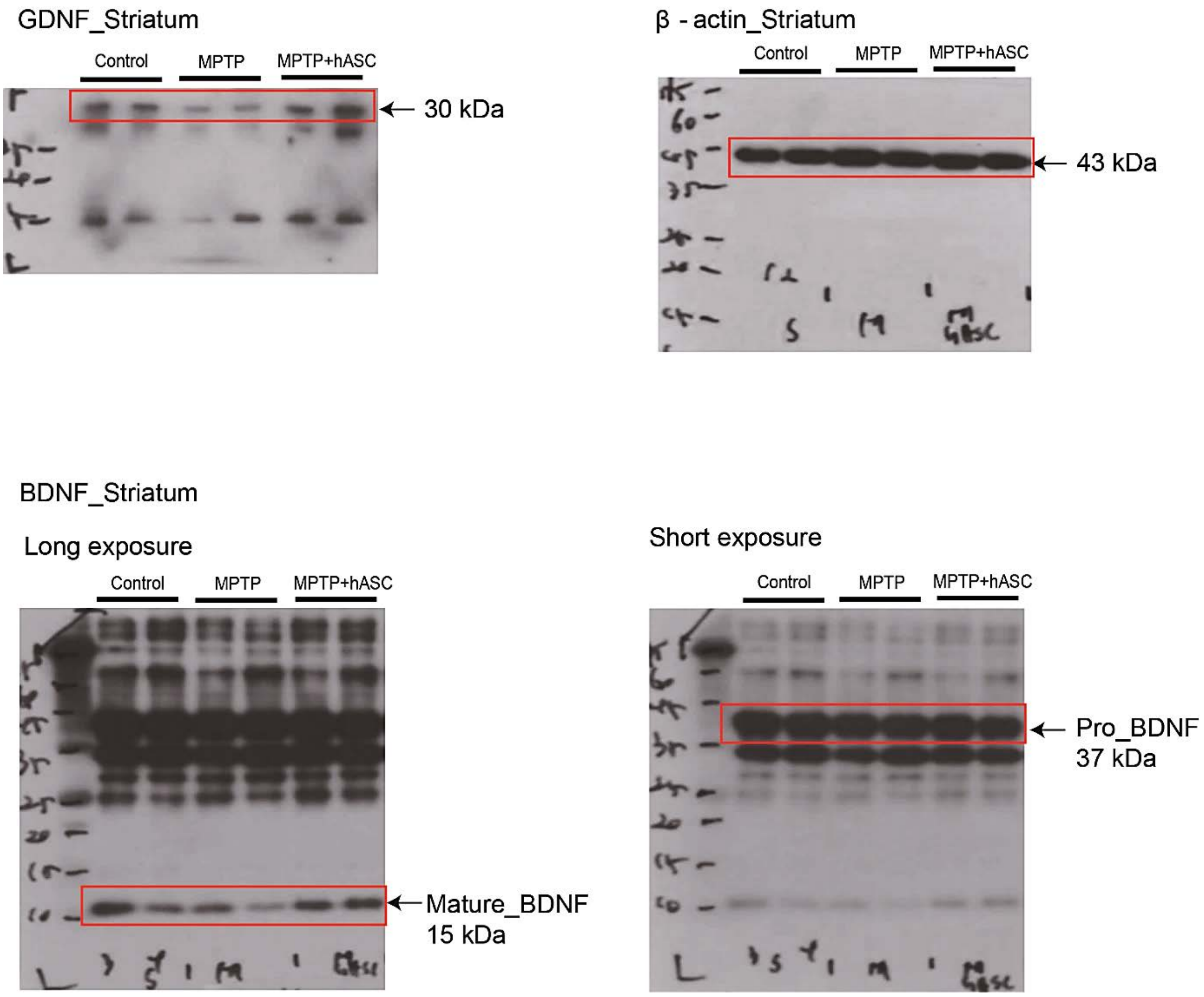

Supplementary Figure 4. Full blots of Figure 4. We read the bands in the red square as GDNF and BDNF normalized by GAPDH.

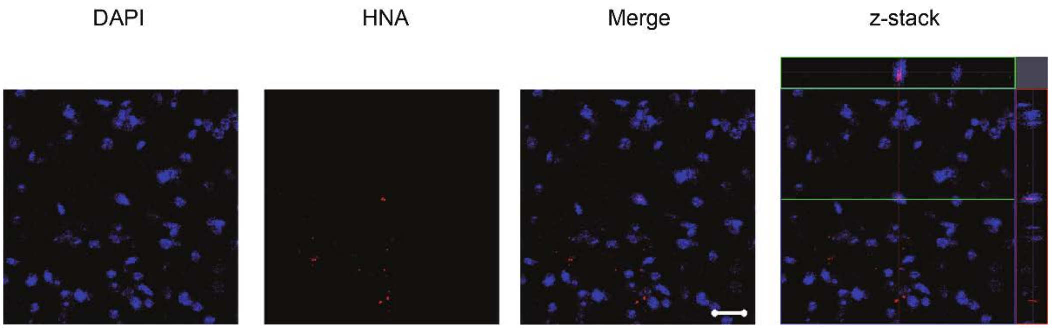

Supplementary Figure 5. The brain slices were immunostained with an HNA antibody to identify transplanted hASCs. Human-specific nuclei in the brain were visualized by Alexa 555 fluorescence for confocal microscope observation. Representative images of TH immunoreactivity in the striatum from mice. Scale bars, 20 μm.
